# Cardiovascular Disease and COVID-19 Among Refugees: A Call to Action

**DOI:** 10.1007/s44197-022-00078-w

**Published:** 2022-11-18

**Authors:** Ahmed Arafa, Rena Kashima, Yoshihiro Kokubo

**Affiliations:** 1 Department of Public Health, Faculty of Medicine, Beni-Suef University, Beni Suef, Egypt; 2 Department of Preventive Cardiology, National Cerebral and Cardiovascular Center, Suita, Japan; 3 Department of Cardiovascular Pathophysiology and Therapeutics, Graduate School of Medicine, Osaka University, Suita, Japan

Dear Editor,

Cardiovascular disease (CVD) remains the leading cause of morbidity and mortality worldwide [1]. The impact of COVID-19 on the CVD burden is a matter of debate and tends to differ from one country to another [2]. However, COVID-19 is closely associated with CVD either directly by elevating the risk of myocardial infarctions, arrhythmias, and strokes or indirectly by creating capacity for COVID-19 patients at the expense of CVD healthcare delivery [2, 3]. CVD, on the other hand, is a major risk factor for COVID-19 severity and mortality [3].

Wars, human rights violations, natural disasters, and economic hardships hitting Africa, the Middle East, Latin America, and South-East Asia in addition to the current state of Ukraine have led to a forcible displacement of tens of millions of people, creating an unprecedented refugee crisis [4]. Unlike the stereotype that the refugee crisis mainly affects developed countries, 80% of international refugees escape a developing country only to settle in another. Among the top ten countries hosting the highest number of international refugees, nine countries are of low or middle income [4], that already experience an increasing CVD incidence [5] and inadequate response to the COVID-19 pandemic [6].

In addition to their suffering in their homelands from violence, persecution, and poverty and their host countries from unhygienic living conditions and discrimination [4], refugees are disproportionately affected by CVD and COVID-19. On the one hand, a meta-analysis of three studies involving refugees residing in the US and Denmark showed that the risk of incident CVD events in refugees was 71% higher than in non-refugee counterparts [7]. The prevalence of traditional CVD risk factors among 331 Cambodian refugees in the US substantially exceeded the corresponding age- and sex-adjusted prevalence among the US population; hypertension (63.6 vs. 47.4%), diabetes (37.8 vs. 16.0%), and hyperlipidemia (73.6 vs. 42.2%) [8]. Further, many refugees suffer from depressive and anxiety symptoms [9] that contribute to CVD development [10]. On the other hand, a retrospective analysis of national surveillance data from Greece showed that COVID-19 outbreaks in 2020 were 2.5 and 2.9 times higher in refugee reception and identification centers than in the general population; most probably due to overcrowding, unsanitary conditions, and limited routine testing and surveillance [11].

Access of refugees to healthcare is mostly restricted and the quality of healthcare they obtain is questionable [12]. Previous studies highlighted significant shortages in providing treatment for refugees, resulting in delays and mortality. For example, out of 196 Syrian refugee children registered for cardiac surgeries at one university hospital in Jordan (184 as a primary procedure and 12 following interventional catheterization), 46 children died (28 preoperatively, 14 in the operative field, and 4 postoperatively) [13]. Another study from Jordan showed that among 969 Syrian refugees with major CVD events, only 322 could receive treatment [14].

Although CVD and COVID-19 are significant challenges to healthcare systems worldwide, these challenges should not be used as an excuse for blocking refugees from obtaining healthcare. Further, the failure to address healthcare inequity among refugees can undoubtedly hinder international efforts to reduce the CVD burden and contain the COVID-19 pandemic [15].

While the refugee crisis is getting worse, several reforms in the healthcare systems of the host countries are needed to include refugees in the national health insurance programs and provide them with health counseling using their languages. Since refugees are at high risk of developing CVD and getting infected with COVID-19, they should be prioritized in CVD risk-prevention programs and COVID-19 vaccination plans. Besides, multi-centric prospective studies targeting refugees are needed to understand the emerging health disorders among this group. Further, providing appropriate preventive and curative health services to refugees is essential to reduce the heavy burden of CVD and COVID-19. These services should be accessible, equitable, and affordable, and refugees should actively participate in deciding the best health services that take into account social and cultural factors.
